# REDD1 Protects Osteoblast Cells from Gamma Radiation-Induced Premature Senescence

**DOI:** 10.1371/journal.pone.0036604

**Published:** 2012-05-18

**Authors:** Xiang Hong Li, Cam T. Ha, Dadin Fu, Mang Xiao

**Affiliations:** Radiation Countermeasures Program, Armed Forces Radiobiology Research Institute, Uniformed Services University of the Health Sciences, Bethesda, Maryland, United States of America; Rush University Medical Center, United States of America

## Abstract

Radiotherapy is commonly used for cancer treatment. However, it often results in side effects due to radiation damage in normal tissue, such as bone marrow (BM) failure. Adult hematopoietic stem and progenitor cells (HSPC) reside in BM next to the endosteal bone surface, which is lined primarily by hematopoietic niche osteoblastic cells. Osteoblasts are relatively more radiation-resistant than HSPCs, but the mechanisms are not well understood. In the present study, we demonstrated that the stress response gene REDD1 (regulated in development and DNA damage responses 1) was highly expressed in human osteoblast cell line (hFOB) cells after γ irradiation. Knockdown of REDD1 with siRNA resulted in a decrease in hFOB cell numbers, whereas transfection of PCMV6-AC-GFP-REDD1 plasmid DNA into hFOB cells inhibited mammalian target of rapamycin (mTOR) and p21 expression and protected these cells from radiation-induced premature senescence (PS). The PS in irradiated hFOB cells were characterized by significant inhibition of clonogenicity, activation of senescence biomarker SA-β-gal, and the senescence-associated cytokine secretory phenotype (SASP) after 4 or 8 Gy irradiation. Immunoprecipitation assays demonstrated that the stress response proteins p53 and nuclear factor κ B (NFkB) interacted with REDD1 in hFOB cells. Knockdown of *NFkB* or *p53* gene dramatically suppressed REDD1 protein expression in these cells, indicating that REDD1 was regulated by both factors. Our data demonstrated that REDD1 is a protective factor in radiation-induced osteoblast cell premature senescence.

## Introduction

More than 50% of cancer patients receive radiotherapy, which often results in side effects due to radiation damage in normal tissue [1]. The hematopoietic system is very sensitive to radiation [2], [3]. Adult mammalian hematopoietic stem and progenitor cells (HSPC) reside in the bone marrow (BM) microenvironment (hematopoietic niche) composed of osteoblast, endothelial and stromal cells. The hematopoietic niche regulates stem cells to self-renew, reproduce, or differentiate into functional blood cells by producing multiple factors and regulating signal transduction. Osteoblast cells constitute a very important niche which supports the maintenance of the BM hematopoietic stem cell (HSC) pool. HSPC and niche cells are implicated in ionizing radiation (IR)-induced BM failure and recovery of niches after IR is essential to HSPC survival [4], [5]. The biological mechanisms of radiation injury including DNA damage, oxidative stress, cell cycle arrest, apoptosis and senescence are now increasingly understood in HSPC, but little is known about the effects of IR on niche cells. Primary cultures of human BM osteoblasts have provided important models to study these cells [6], [7]. However, the scarcity, heterogeneity, and limited cell number and lifespan of primary cell cultures restrict their usefulness [8], [9]. In an effort to overcome these limitations, a conditionally immortalized human fetal-osteoblast cell line, human fetal osteoblast 1.19 (hFOB), was established [10] and many studies, including ours, have been reported using this cell line [11]–[14]. hFOB cells possess similar cell surface marker as human bone marrow mesenchymal stromal cells [14] and can form bone *in vivo* and extracellular matrix *in vitro* without developing cell transformation [12], suggesting a good model for the study of osteolineage cell biology in vitro. In the present study we showed that γ radiation induced premature senescence in hFOB cells, and a stress response gene REDD1 (regulated in development and DNA damage responses 1, also known as RTP801, DDIT4 and Dig-1) [15], [16] was highly expressed in hFOB cells after γ radiation.

Previous studies demonstrated that REDD1 is a transcriptional target of p53 [15], [17] and plays a bi-functional role as a pro-survival or pro-apoptotic factor in different type of cells in response to different stressors [16]. In addition, REDD1 is a crucial inhibitor of mammalian target of rapamycin (mTOR) which regulates cell growth in response to environmental inputs [18]. However, the effects of REDD1 in IR-induced intracellular signaling are not well understood. In the present study, we demonstrated that REDD1 inhibited mTOR and the cyclin-dependent kinase inhibitor p21 in γ-irradiated hFOB cells and protected these cells from stress-induced premature senescence (SIPS). Recent reports suggested that the mTOR pathway is involved in cellular senescence [19]–[21]. Under environmental stress, cells rapidly activate a variety of adaptive mechanisms that limit energy expenditure through inhibition of energy-intensive processes to protect important functions such as DNA production and repair. However, in some types of cells, radiation or DNA damage-induced cell cycle arrests do not inhibit Ras/AKT/mTOR growth-promoting pathways but often activate them [20]. When the cell cycle is inhibited but mTOR is not, the cells become senescent [20]. REDD1-inhibited p21 expression and mTOR signaling may contribute to survival of irradiated hFOB cells through energy-saving [22], [23] and anti-senescence mechanisms. Furthermore, our data show that IR-induced REDD1 expression is regulated by p53 and nuclear factor κB (NFkB). Tumor suppressor protein p53 is a key player in response to IR-induced cell damage [24], and p53 and NFkB have major roles in the regulation of cellular senescence [25]. In this study, the interaction of REDD1 and stress response factors including p53, NFkB, replication protein A2 (RPA2) and their downstream factors mTOR and p21 in response to radiation were elucidated.

## Results

### Gamma Radiation-induced Apoptosis and Senescence in hFOB Cells

hFOB cells were exposed to γ radiation (0, 4, or 8 Gy at a dose rate of 0.6 Gy/min) according to our previous reports [13], and flow cytometry apoptosis assays using Annexin-V (apoptotic cell marker) and 7-aminoactinomycin D (7AAD, a death marker) staining were performed 24 and 48 h later. hFOB cells displayed no increase in apoptotic cells at 24 h after a dose of 4 and 8 Gy, and the percentages of Annexin-V- and 7AAD-positive cells slightly increased from 6.2±3.8% (0 Gy control) to 13.6±4.7% at 48 h after 8 Gy (Figure S1A). To confirm the flow cytometry analysis, an adenosine triphosphate (ATP) survival assay was performed using a bioluminescence method. The concentration of ATP is stable in live cells. Surprisingly, radiation did not affect ATP levels in hFOB cells within 48 h after IR compared to non-irradiated controls, as shown in Figure S1B.

**Figure 1:**
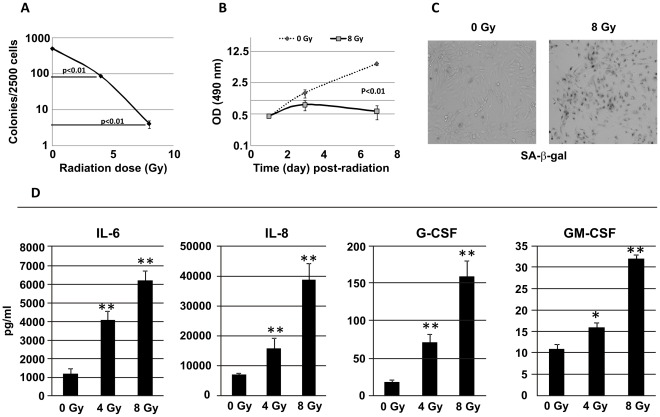
Gamma-radiation induced senescence in hFOB cells. Following radiation (0, 4, or 8 Gy), senescent hFOB cells were determined by (A) clonogenicity assays, (B) MTS-proliferation assay and (C) SA-β-gal staining. SA-β-gal-positive cells were increased 72 h after 8 Gy γ irradiation. Representative data was from one of a total of 3 independent experiments. (D) Conditioned medium (CM) from hFOB cells was collected and pooled from three experiments 48 h after irradiation and the concentration of cytokines was analyzed by Luminex in triplicate. Gamma radiation-induced IL-6, IL-8, G-CSF and GM-CSF were significantly increased. Means ± SD. *: p<0.05, **: p<0.01, IR vs. non-irradiated cells.

We next evaluated senescence in γ-irradiated hFOB cells. Senescent cells display a number of characteristics including cell cycle arrest and irreversible loss of proliferative potential and activation of the senescence-associated beta-galactosidase (SA-β-gal) activity, and exhibit a senescence-associated cytokine secretory phenotype (SASP) [26]. Colony formation assays were performed immediately after sham- or γ irradiation in hFOB cells and colonies were scored 14 days later. As shown in Figure 1A, clonogenicity of hFOB cells was significantly inhibited by 4 and 8 Gy irradiation in a radiation dose-dependent manner. Cell proliferation potential was also measured using the CellTiter 96R AQueous non-radioactive cell proliferation assay (MTS-assay). The quantity of formazan product as measured by the amount of 490 nm absorbance (OD) is directly proportional to the number of living cells in culture. Levels of proliferation as shown by OD were dramatically low in 8 Gy irradiated samples compared with control (Figure 1B; p<0.01). Consistently, SA-β-gal activity was increased in hFOB cells 72 h after 8 Gy irradiation (Figure 1C). Furthermore, γ radiation-induced cytokine secretion was examined using the multiplex Luminex assay [27] as described in Materials and Methods. Previous reports indicated that proinflammatory cytokines such as interleukin (IL)-6 and IL-8 are essential, in a cell-autonomous fashion, for cells to enter senescence [28]. In the present study we examined IL-6, IL-8, granulocyte colony-stimulating factor (G-CSF), and granulocyte-macrophage colony-stimulating factor (GM-CSF) secretion from 0, 4, and 8 Gy- irradiated hFOB cells. hFOB cell conditioned medium (CM) was collected 48 h post-irradiation and samples were pooled from three independent experiments before analysis. Concentrations of cytokines in these samples were measured in triplicate. As shown in Figure 1D, levels of IL-6, IL-8, G-CSF and GM-CSF were remarkably increased in CM from irradiated hFOB cells in a radiation dose-dependent fashion. This radiation-associated cytokine secretion together with cell proliferation inhibition and SA-β-gal activity confirmed that γ radiation induced hFOB cell senescence.

### Gamma Radiation Induced REDD1 Expression in hFOB Cells

**Figure 2:**
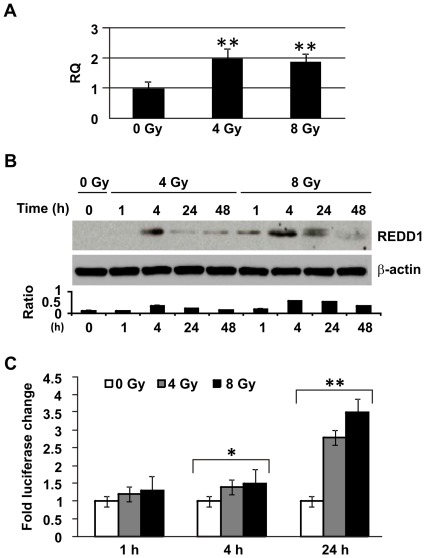
Radiation-induced REDD1 expression in hFOB cells. (A) Quantitative RT-PCR, mRNA levels for *REDD1* in hFOB cells using 18 S rRNA as a control to calculate the relative quantity (RQ) of gene expression 4 h after IR (0, 4, or 8 Gy). (B) Western blot determined REDD1 protein levels in hFOB cells at the indicated time points after different doses of radiation. Representative immunoblots and ratios of REDD1/β-actin from three experiments are shown. (C) hFOB cells were transiently transfected with PGL3-basic luciferase-REDD1 promoter (−2548/+166bp) construct or PGL3-basic luciferase-vector (control). β-galactosidase was used to delineate transfection efficiency. At 16 h after transfection, cells were exposed to 0, 4 or 8 Gy γ radiation. Cells were harvested at 1 h, 4 h and 24 h post-IR. Luciferase activity was determined, and the fold changes of luciferase activity are shown. Results are from a total of three experiments. Means ± SD. *: p<0.05, **: p<0.01, IR vs. non-irradiation (0 Gy) Means ± SD.

We previously demonstrated that γ radiation activated multiple stress response proteins in hFOB cells including p53, p21, p38, c-Jun and NFkB [13]. REDD1 is a transcriptional target of p53 and expressed ubiquitously in mammalian cells [15], [17]. To evaluate the effects of REDD1 on radiation-induced senescence, we determined REDD1 expression in hFOB cells in response to γ radiation. REDD1 mRNA expression in response to radiation was very transient. Quantitative RT-PCR showed two-fold increases of *REDD1* mRNA in both 4 and 8 Gy-irradiated hFOB cells only at 4 h post-IR (Figure 2A). The levels of REDD1 protein expression in hFOB cells were increased from 4 h to 48 h after γ irradiation in a radiation dose-dependent manner (Figure 2B). Forty-eight h after irradiation, levels of REDD1 protein had decreased and returned to baseline levels as shown in non-irradiated control. The peak of REDD1 expression occurred 4 h post-IR in both 4 and 8 Gy-irradiated cells.

To confirm whether or not *REDD1* promoter function is regulated by γ radiation in hFOB cells, we analyzed transcriptional activity of the promoter. The 2.5 kb *REDD1* promoter (−2548 to +166 bp) was fused with a firefly luciferase reporter gene [29]. This construct was transiently transfected into hFOB cells and treated with 4 or 8 Gy γ radiation. As shown in Figure 2C, γ irradiation induced *REDD1* promoter activation in a dose and time-dependent manner. 8 Gy radiation activated the promoter 3.5-fold at 24 h post-irradiation, compared to non-irradiated controls. Forty-eight h after irradiation, the promoter activation levels had returned to baseline levels observed in non-irradiated control (data not shown).

### REDD1 Protects hFOB Cells from γ Radiation-induced Senescence

**Figure 3:**
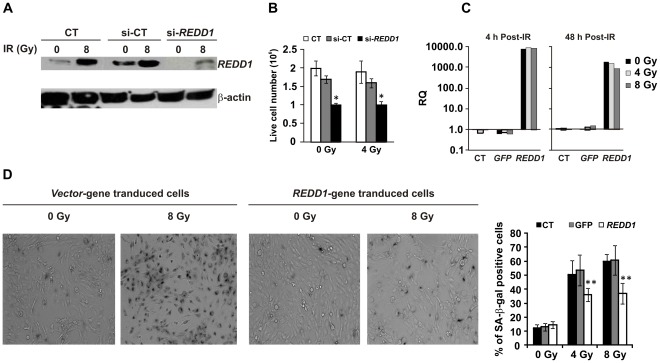
REDD1 protects hFOB cells from γ radiation-induced senescence. The effects of REDD1 were evaluated using gene silencing (si-RNA) and overexpression (plasmid DNA transfection) approaches. (A) Western blot shows REDD1 and β-actin (loading control) expression in control (non-transfection CT), REDD1 siRNA-transfected (si-REDD1), and maxGFP siRNA-transfected control (si-CT) samples after 0 or 8 Gy irradiation. Transfection of si-REDD1 decreased the radiation-induced REDD1 expression. (B) Survival cell number (trypan blue–negative) was decreased in siREDD1-transfected hFOB cells 24 hours after IR. Means ± SD for 3 independent experiments. *: p<0.05, si-REDD1 vs. CT and si-CT. (C) Quantitative RT-PCR determined *REDD1* gene expression in non-gene transfected control (CT), vector-transfected(GFP) and REDD-gene containing construct transfected hFOB cells at 4 and 48 h after irradiation. The relative quantity of gene expression was calculated using 18 S rRNA as a control. (D) Overexpression of REDD1 inhibited SA-β-gal activation after irradiation. Representative image of SA-β-gal staining and statistical data from three experiments are shown. Means ± SD. **: p<0.01, REDD1 plasmid DNA-transfected vs. CT or vector-transfected samples.

To investigate the role of endogenous REDD1 in irradiated hFOB cells, we examined the effects of silencing REDD1 expression. REDD1 siRNA was transfected into hFOB cells before γ irradiation using nucleofector technology [13]. Cells were exposed to γ radiation 24 h after transfection and REDD1 protein expression was examined 4 h post-IR (28 h after siRNA transfection). Immunoblotting assays (Figure 3A) showed that REDD1 protein levels markedly decreased after REDD1 siRNA transfection in both irradiated and non-irradiated cells. In contrast, control siRNA transfected cells expressed REDD1 at the same level as nontransfected samples. Knockdown of REDD1 resulted in a decrease in live cell number from 1.7±0.2×10^6^ (control-siRNA) to 1±0.08×10^6^ (REDD1-siRNA) 24 h after IR (48 h after siRNA transfection) and the live cell number decreases were independent of irradiation (p<0.05, REDD1-siRNA vs. control-siRNA, Figure 3B).

**Figure 4:**
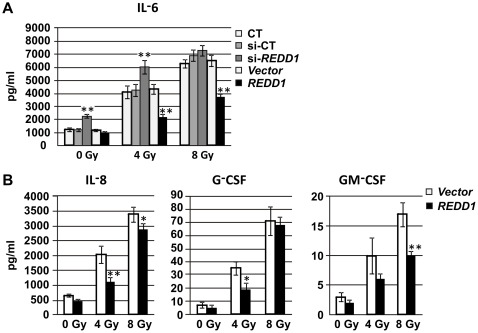
REDD1inhibits senescence-associated cytokine secretory phenotype (SASP) in irradiated hFOB cells. Conditioned medium (CM) from hFOB cells were pooled from three independent experiments, the concentration of cytokines was analyzed by Luminex in triplicates. (A) Effects of REDD1 on IL-6 secretion were further evaluated in hFOB cells with si-REDD1 gene or REDD1 plasmid DNA transfection and irradiation. Means ± SD. **: p<0.01, REDD1siRNA or REDD1 plasmid DNA-transfected vs. CT or si-CT or vector-transfected samples. (B) Overexpression of REDD1 decreased levels of IL-8, G-CSF and GM-CSF in irradiated hFOB cell CM. Means ± SD. *: p<0.05, **: p<0.01, REDD1 plasmid DNA-transfected vs. vector-transfected samples.

We next transfected REDD1 plasmid DNA construct into hFOB cells. PCMV6-AC-GFP (vector control) or PCMV6-AC-GFP-REDD1 plasmid DNA (purchased from OriGENE Bethesda, MD) was transiently transfected into hFOB cells before γ irradiation using FuGENE 6 reagent. Cells were exposed to γ radiation 24 h after transfection and REDD1 mRNA and protein expression were examined post-IR. Over 1000-fold increases in *REDD1* mRNA expression were shown by Quantitative RT-PCR (Figure 3C) at 4 and 48 h post-IR (72 h after gene-transfection) in REDD1 plasmid DNA transfected vs. control DNA transfected cells. Overexpression of REDD1 significantly suppressed frequency of SA-β-gal-positive cells in both 4 and 8 Gy irradiated samples and the SA-β-gal-positive senescent cells decreased from 62±11% to 35±7% in 8 Gy irradiated hFOB cells as shown in Figure 3D (p<0.01). We further measured effects of REDD1 on SASP of irradiated hFOB cells. Overexpression of REDD1 significantly inhibited radiation-induced IL-6 secretion, whereas knockdown of REDD1 caused an increase in IL-6 in hFOB cell conditioned medium even without IR (Figure 4A). IL-8, G-CSF and GM-CSF secretion after IR also inhibited by REDD1 overexpression (Figure 4B), suggesting the senescence-suppression effect of REDD1 in these cells.

### IR Induced REDD1 Signal Transduction Cascades

**Figure 5:**
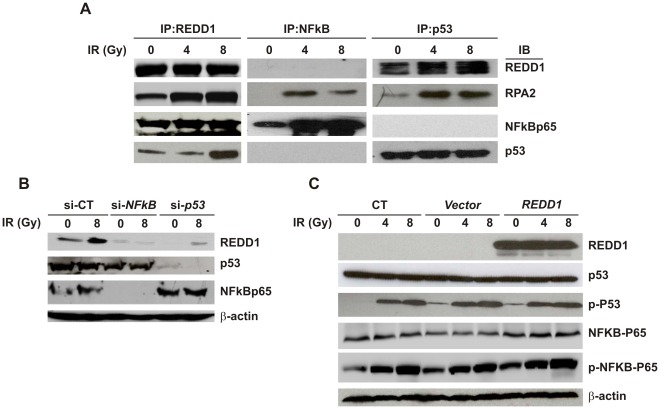
IR induced REDD1 signal transduction cascades. (A) hFOB cell lysates collected at 4 h after IR were subjected to immunoprecipitation using REDD1, NFkBp65 or p53 antibodies, respectively. After SDS-gel separation, proteins were analyzed by immunoblotting using anti-REDD1, NFkBp65, RPA2 and p53 antibodies. (B) Western blot using hFOB cell lysates shows REDD1, p53, NFkBp65 and β-actin (loading control) expression in control, NFkBp65-siRNA-transfected, and p53-siRNA-transfected samples. Knockdown of either NFkBp65 or p53 gene resulted in attenuated REDD1 protein expression. (C) Western blot shows REDD1, p53 and NFkBp65 expression and p53 (serc15) and NFkBp65 (ser 536) phosphorylation in control, vector or REDD1 plasmid DNA-transfected samples. Overexpression of REDD1 had no effects on p53 and NFkB expression and activation. Representative immunoblots from three experiments are shown.

Gamma radiation-induced p53 and NFkB activation in hFOB cells has been reported by us [13]. In this study, using immunoprecipitation (IP) assays, we demonstrated the interaction of p53, NFkBp65 and replication protein A 2 (RPA2) with REDD1 in hFOB cells. Figure 5A shows that after IP using anti-REDD1 antibody to pull down REDD1 from cell lysate, RPA2, NFkBp65 and p53 protein expression was detected. IP with anti-NFkBp65 antibody only captured RPA2. When IP with anti-p53 antibody was performed, REDD1 and RPA2 but not NFkBp65 were detected. Interestingly, RPA2 associated with all three proteins and its interactions with either REDD1 or NFkB or p53 were radiation-dependent. Knockdown of NFkBp65 or p53 by siRNA significantly suppressed REDD1 protein expression (Figure 5B), indicating that REDD1 was regulated by both factors. Furthermore, transfection of REDD1 plasmid DNA dramatically increased REDD1 protein levels in hFOB cells. However, it did not influence expression or phosphorylation of p53 or NFkB in response to IR (Figure 5C). REDD1, p53 and NFkB did not appear to be direct upstream regulators or downstream targets of RPA2 (data not shown).

**Figure 6:**
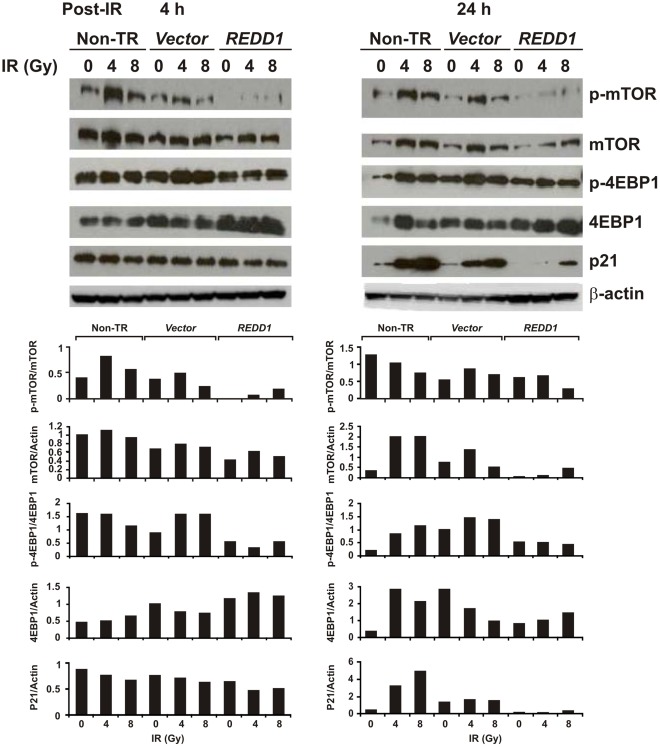
Overexpression of REDD1 suppressed mTOR and p21 expression, and inhibited mTOR activation. Western blot and data summaries show mTOR, 4EBP1 and p21 expression and phosphorylation of mTOR and 4EBP1 in non-gene transfected control (Non-TR), vector or REDD1 plasmid DNA-transfected samples 4 and 24 h after irradiation. Representative immunoblots and indicated ratios from three experiments are shown.

We next tested downstream targets of REDD1 in irradiated hFOB cells. Immunoblotting assays (Figure 6) demonstrated that over-expression of REDD1 inhibited mTOR expression and phosphorylation, and moderately downregulated the phosphorylation of its downstream target eukaryotic initiation factor 4E (eIF4E)-binding protein-1 (4EBP-1) at 4 and 24 h after irradiation. The expression of the cyclin-dependent kinase inhibitor p21 also was inhibited 24 h post-irradiation.

## Discussion

Ionizing radiation (IR) induces DNA double strain breaks (DSB) in mammalian cells, with subsequent cell cycle arrest, apoptosis and/or senescence, depending on the cell type and stage of development. We demonstrated that features of the response of hematopoietic stem and progenitor cells and hematopoietic niche osteoblast cells to γ radiation are different. IR caused death of primary human hematopoietic CD34+ cells through apoptosis [30], whereas it induced senescence in human fetal osteoblast cell line cells. The stress-induced senescent cell markers, including proliferative (clonogenicity)-inhibition, SA-β-gal activity and senescence-associated secretory phenotype (SASP) were markedly displayed in hFOB cells after 4 and 8 Gy γ irradiation. In contrast, indications of radiation-induced apoptosis in hFOB cells (S1) were relatively lower than in CD34+ cells [13]. Furthermore, we found that a novel cell stress response gene REDD1 [15], [16] is highly induced in mouse bone marrow osteoblastic cells (unpublished data) and hFOB cells in response to γ radiation. The molecular mechanisms of REDD1 were further studied in hFOB cells. Knockdown of REDD1 by siRNA resulted in hFOB cell number decreases. In contrast, over-expression of REDD1 inhibited mTOR and p21 expression, suppressed inflammatory factor secretion and senescent cell marker SA-β-Gal activity in hFOB cells, and protected these cells from γ radiation-induced senescence (Figure 3). It has been suggested that the tumor suppressor protein p53 and its downstream p21 and p16 signal transduction cascades in human cells mediate the activation of the senescence program, and therefore have been used as biomarkers to identify senescent cells [26]. In general, activation of p53 and its downstream signaling molecule p21 in cells undergoing senescence occurred prior to the expression of p16. Overexpression of REDD1 inhibited p21 expression in irradiated hFOB cells, which confirmed its anti-senescence and host defense effects in these cells. At present, how REDD1 inhibited p21 expression is not clear. However, p53 has bind site in the promoter region of p21 [31] and REDD1 [15] gene and regulates their expression. According to Hill et al. [31], the nature of DNA damage enables p53 to selectively discriminate between promoters in the induction of target genes, thereby regulating their expression and subsequent cellular outcome. Whether overexpressed REDD1 inhibits transcriptional activity of p53 on p21 gene or enhances p21 protein degradation are under investigation.

Furthermore, our study showed that overexpression of REDD1 in hFOB cells suppressed mTOR and phosphorylation of its downstream target 4EBP-1 (mTOR signal inhibitor); this suggests inhibition of radiation-induced mTOR signal pathway activation. mTOR is a key protein kinase that regulates cell growth and metabolism to maintain cellular and organismal homeostasis. Braunstein et al [32], suggested that early, transient mTOR-induced cap-dependent mRNA translation after IR contributed to DNA repair and cell survival. Our recent study confirmed the protective effect of mTOR on γ radiation-induced apoptosis in human hematopoietic CD34+ cells and mouse hematopoietic cells [33]. However, recent studies from Demidenko et al. [19], [34], [35] demonstrated the mTOR pathway is involved in cellular senescence. Their hypothesis is that when the cell cycle is inhibited by stress (such as radiation or DNA damage), induction of p53 and its downstream target p21 inhibit cell proliferation. However, if mTOR is still active as a result of stress-induced growth factor secretion, it will cause cell hypertrophy and senescence [19], [35]. Eventually, lysosomal enzymes, such as β-D-galactosidase activity, will result in the senescent cell’s lysosomal membrane breakdown and the release of lysosomal proteases into the cytosolic compartment. Blocking of the mTOR activity and hypophosphorylation of 4EBP1 before radiation-induced cellular senescence commences is necessary for saving energy and for assembly of the DNA damage response machinery [32]. Hence REDD1, as an essential negative regulator of mTOR [22], [18], may play an important role in suppressing mTOR-induced protein synthesis [23] and cell senescence.

We further investigated REDD1 regulation in irradiated hFOB cells. Immunoprecipitation assays demonstrated that the stress response proteins p53, RPA2 and NFkB were associated with REDD1 in hFOB cells. Knockdown of *NFkB* or *p53* gene by siRNA dramatically suppressed endogenous REDD1 protein expression in irradiated hFOB cells, indicating that REDD1 was regulated by both factors. Moreover, overexpression of REDD1 did not change expression and phosphorylation of p53 or NFkB after irradiation, suggesting their activation is REDD1- independent. The stress-activated p53 and NFkB signaling pathways are key players in the regulation of cellular senescence and organismal aging [25]. Accumulated evidence has indicated that p53 signaling is functionally antagonistic to the NFkB system. However, the tumor suppressor p53 is an important trigger of cellular senescence and NFkB signaling is involved in the induction of the SASP. Interestingly, we report for the first time that REDD1 expression is regulated by both p53 and NFkB simultaneously. Whether REDD1 inhibition of SASP is NFkB-dependent, or whether there is a feedback loop which results in REDD1 inhibition of NFkB activation, needs further study. Furthermore, the interaction of REDD1 and RPA2 in irradiated hFOB cells supports the survival-promoting role of REDD1 in these cells. RPA, the primary single-stranded DNA (ssDNA) binding protein, is indispensible for DNA repair (including SSBs and DSBs) and replication after DNA damage in eukaryotes. RPA is a heterotrimer composed of 70 kDa (RPA1), 32 kDa (RPA2), and 14 kDa (RPA3) subunits [36]. RPA2 is hyperphosphorylated after exposure to radiation [37] through ATM and DNA-PK regulation, and is preferentially recruited to DSB repair in a checkpoint-dependent manner. p53 and RPA complexes after DNA damage are linked with DNA repair and p53-dependent checkpoint control [38]. Our data are consistent with this model and suggest that REDD1 may be involved in p53 and RPA survival signaling in response to IR.

Our data also show that endogenous REDD1 was expressed at 4 to 48 h after IR, with peak expression at 4 h in osteoblast cells. This p53- and NFkB-induced expression of REDD1 at a relatively early stage of the response to IR could inhibit p21 and mTOR activation and protect cells from senescence. Previous reports suggested that p53 can suppress senescence through inhibition of mTOR [35], [39], [40]. Our data further suggest that the effect of p53 on inhibition of mTOR may be through upregulation of REDD1 in irradiated hFOB cells. This hypothesis is under investigation.

In conclusion: REDD1 is regulated by p53 and NFkB signaling in response to radiation and plays an important role in suppressing p21- induced cell proliferation arrest and mTOR-induced protein synthesis, hence protects osteoblast cells from radiation-induced premature senescence.

## Materials and Methods

### Cells and Gamma-irradiation

The human fetal osteoblast cell line (hFOB 1.19) [14] was obtained from the American Type Culture Collection (ATCC, Manassas, VA, USA) and cultured following the ATCC protocol [13]. hFOB cells were cultured in a 1∶1 mixture of phenol-free Dulbecco’s modified Eagle’s medium/Ham’s F-12 medium (DMEM-F12, Invitrogen, Carlsbad, CA), supplemented with 10% fetal bovine serum (FBS) (Hyclone, Logan, UT), 2 mM L-glutamine, and antibiotics. Cells were incubated at 34°C with 5% CO_2_.

hFOB cells were irradiated at doses of 0, 4 or 8 Gy (0.6 Gy/min) in the Armed Forces Radiobiology Research Institute Cobalt facility, according to our previous reports. [13], [33].

### Apoptotic Flow Cytometry and Colony Forming Assay

Cell expansion and viability (trypan blue-negative cells) from all groups were counted. Labeling with a cell death marker 7-aminoactinomycin D (7AAD) and an apoptotic marker (Annexin-V) was determined using BD FACSCalibur flow cytometry. All antibodies and dyes were purchased from BD Biosciences (San Jose, CA, USA).

hFOB cells were seeded with 1000–5000 cells/well in 6-well plates in DMEM-F12 complete medium with 10% FBS for clonogenic survival assays in triplicate. After 14 days of incubation, cells were fixed with methanol and stained with crystal violet, and colonies with more than 50 cells were scored.

### ATP-assay and SA-β-gal Staining

ATP-assays were performed using the Enzylight™ ATP Assay kit (EATP-100, BioAssay System, Hayward CA) according to the manufacturer’s protocol. In briefly, plate cells at 100 µL/well in white opaque tissue culture plates, 5 µL of test compounds and controls dissolved in PBS per well were added. Plates were rocked and incubated overnight. For each test well, 95 µL Assay Buffer was mixed with 1 µL Substrate and 1 µL ATP Enzyme. 90 µL reconstituted reagent was added to each test well and mixed by tapping the plate. After incubating for 2 minutes at room temperature, luminescence was read on a luminometer (Berthold Luminometer), with an integration time of 0.1 to 5 sec.

Beta-galactosidase (β-gal) was assayed using a kit from abcam Inc. (Cambridge, MA). Cells were fixed for 5 min in β-galactosidase fixative (2% formaldehyde; 0.2% glutaraldehyde in PBS), and washed with PBS and stained in β-galactosidase fixative solution (1 mg/ml 5-bromo-4-chloro-3-indolyl-beta-gal in 5 mM potassium ferricyamide, 5 mM potassium ferrocyamide, 2 mM MgCl2 in PBS) at 37°C until β-gal staining became visible in either experimental or control plates. Cells were washed in PBS, and the numbers of β-gal-positive cells (blue staining) in at least 200 cells were counted in random fields in each of the triplicate wells.

### Cell Proliferation Assay (MTS-assay)

MTS [3-(4, 5-dimethylthiazol-2-yl)-5-(3-carboxymethoxyphenyl)-2-(4-sulfophenyl)-2H-tetrazolium, inner salt) is bioreduced by cells into a formazan product that is soluble in tissue culture medium. The quantity of formazan product as measured by the amount of 490 nm absorbance (OD) is directly proportional to the number of living cells in culture. MTS-assays were performed using the CellTiter 96^R^ AQueous Non-Radioactive Cell Proliferation Assay kit (Promega) according to the manufacturer’s protocol. In brief, after irradiation, hFOB cells were plated at 5000 cells/well of 96 well plate in quadruplet. At the indicated times, 20 µl of MTS/PMS solution (ratio 20/1) was prepared and added to the wells containing a final volume of 100 µl medium. The plates were incubated for 4 hours at 37°C and the OD at 490 nm was recorded using an ELISA plate reader. The average 490 nm absorbance from three “no cell” control wells was used as blank.

### Quantitative Real-time PCR (QRT-PCR)

Total RNA was extracted from 5×10^5^ cultured hFOB cells using RNAqueous-4PCR Kits from Ambion (Austin, TX, USA) and was reverse-transcribed using random hexamers according to the manufacturer’s instructions (Bio-Rad, Hercules, CA, USA). Multiplex QRT-PCR assays were carried out as described in our previous report [30]. Human REDD1 PCR primers and probe (Biosearch Technologies, Inc, 81 Digital Drive, Novato, CA 94949-5728) sequences were as follows:

Forward primer: 5′- GCC AGG TGG GCA AAG AAC -3

Reverse primer: 5′- CAC GCT GTG GCA GCT CTT G -3′

Probe: 5′ Quasar 670-TACTGCGCCTGGCCTACAGC-3′BHQ2

### siRNA and Plasmid DNA Transfection

REDD1, NFkBp65, and p53 siRNA from siGENOME SMARTpool (Dharmacon Inc., Lafayette, CO) were transfected into hFOB cells using a Nucleofector II (amaxa Inc., Gaithersburg, MD) according to the manufacturer’s protocol. In brief, 10^6^ hFOB cells were resuspended in 100 µl of human cell Nucleofector solution with 1.5 µg of siRNA-siGENOME SMARTpool and/or 1.5 µg of maxGFP siRNA (positive control provided in the siRNA Test Kit, amaxa, Inc) using program A-27 as discussed in our previous report [30].

hFOB cells (1.45 million cells per 10 cm dish) were transfected with PCMV6-AC-GFP or PCMV6-AC-GFP-REDD1 plasmid DNA (11 µg/dish) from OriGene (Rockville, MD) using FuGENE 6 reagent (35 µl/dish) according to the manufacturer’s protocol (Roche). 24 h after transfection, cells were subjected to 0, 4 and 8 Gy IR. Cells were harvested at 4, 24 and 48 h post-IR for further analysis.

### Immunoprecipitation (IP) and Immunoblotting (IB)

IP kits from Sigma (Saint Louis, Missouri) were used. 1–5×10^6^ cells from each sample were harvested, washed, and lysed with 0.5 ml lyses buffer, 1–5 µg of purified primary antibody, 1x IP buffer (provided in kit), and protease inhibitor cocktail. Components were added to a spin column and incubated overnight at 4°C with inversion. Precleared protein G beads (20–30 µl) were added to the column and incubated overnight at 4°C. After wash, 50 µl 1x Laemmli sample buffer was added to the pellet. After being vortexed and heated to 90–100°C for 5 min, samples were spun at 10,000 g for 5 min, and supernatants were collected for SDS-PAGE. IB was performed following standard procedures with an enhanced chemiluminescence kit (Thermo Scientific, Rockford, IL) and Kodak X-ray film or Fuji image. Antibodies for REDD1 were from ProteinTech (Chicago, IL); p53, NFkB-p65 and p21 were from Santa Cruz Biotechnology, Inc., (Santa Cruz, CA); phospho (p)-p53, p-NFkB-p65, mTOR, and p-mTOR were from Cell Signaling (Danvers, MA): RPA2 was from abcam Inc. (Cambridge, MA).

### Promoter Activity Assay

hFOB cells were cotransfected with PGL3-basic luciferase-vector (Promega) or PGL3-basic luciferase-REDD1 promoter (generous gift from Y. Chen, Indiana University, Indianapolis, IN ) [29] and β-galactosidase expression vector as an internal control for transfection efficiency (8 µg PGL3, 8 µg PGL3-REDD1 promoter and 0.6 µg B-galactosidase). 16 h after transfection, cells were subjected to 0, 4 or 8 Gy γ irradiation. Cells were harvested at 1 h, 4 h, 24 h and 48 h post-IR. Luciferase activity was determined using the Luciferase Assay System (Promega Corp., Madison, WI). To normalize for transfection efficiency, β-galactosidase activity was determined in the same cell extracts using the Galacto-Light Plus Systems (Applied Biosystems, Bedford, MA).

### Analysis of Cytokines by *Luminex*

Cytokine detection was performed using the Luminex-100 (Luminex Corp, Austin, TX, USA) [27]. Conditioned medium (CM) from hFOB cells were pooled from three independent experiments and were diluted according to protein concentration measurement (using a bicinchoninic acid protein assay kit, Pierce, Rockford, IL, USA). Samples were pipetted into the wells of a filter bottom microplate. Cytokine antibody-conjugated microspheres were added to each well, incubated and washed. Then diluted biotinylated antibody (R & D Systems, Inc., Minneapolis, MN, USA) was added. After incubation and removal of excess biotinylated antibody, streptavidin-phycoerythrin (Molecular Probes, Inc., Eugene, OR, USA) was added. After final incubation and washing, the fluorochrome bound to microspheres was quantified and was directly proportional to the concentration of cytokine.

### Statistical Analysis

Differences between means were compared by ANOVA and Student’s *t* tests. *P<*0.05 was considered statistically significant. Results are presented as means ± standard deviations of the mean as indicated.

## Supporting Information

Figure S1 **Gamma radiation-induced apoptotic cell death in irradiated hFOB cells.** hFOB cells were subjected to 4 and 8 Gy irradiation. (A) Flow cytometric analysis for the apoptotic cell death marker Annexin-V/7AAD 48 h after irradiation. Representative data from three experiments are shown. (B) Intracellular ATP levels were evaluated in hFOB cells at different times after irradiation. Results are from a total of three experiments. No significant changes in ATP level were observed in sham- and γ-irradiated cells.
(TIF)
