# Supplementary figures and images for: REDD1 Protects Osteoblast Cells from Gamma Radiation-Induced Premature Senescence
Source: PLoS One. 2012 May 18;7(5):e36604. doi: 10.1371/journal.pone.0036604 (PMC3356368; doi:10.1371/journal.pone.0036604)

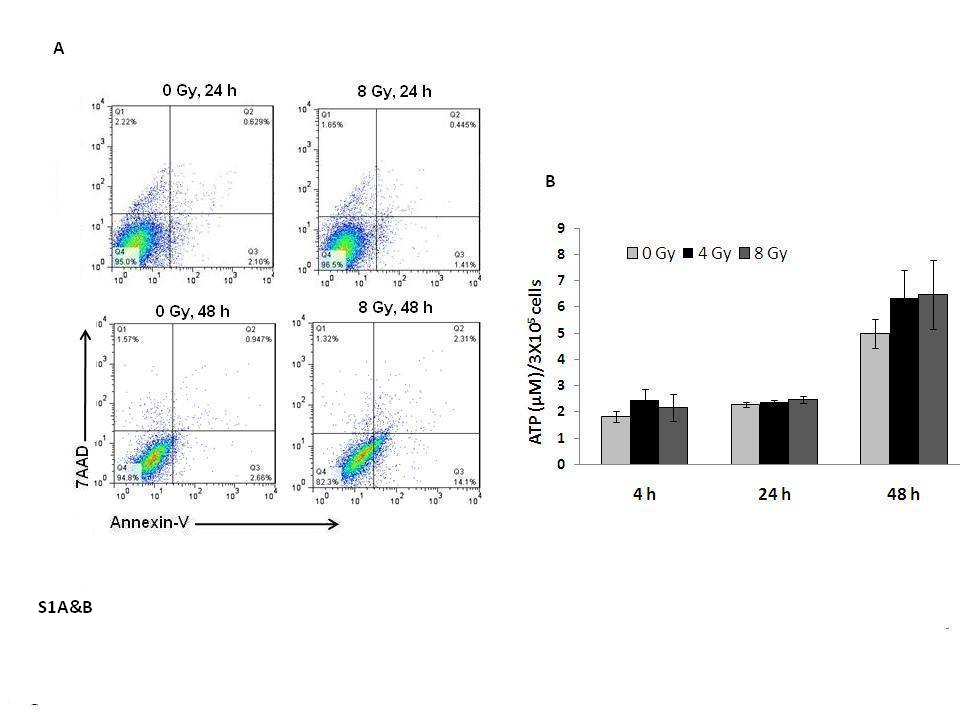

Supplement: Figure S1 — Gamma radiation-induced apoptotic cell death in irradiated hFOB cells. hFOB cells were subjected to 4 and 8 Gy irradiation. (A) Flow cytometric analysis for the apoptotic cell death marker Annexin-V/7AAD 48 h after irradiation. Representative data from three experiments are shown. (B) Intracellular ATP levels were evaluated in hFOB cells at different times after irradiation. Results are from a total of three experiments. No significant changes in ATP level were observed in sham- and γ-irradiated cells. (TIF) [file pone.0036604.s001.tif]
